# A phase II dose-escalation trial of perioperative desmopressin (1-desamino-8-d-arginine vasopressin) in breast cancer patients

**DOI:** 10.1186/s40064-015-1217-y

**Published:** 2015-08-19

**Authors:** Ruth S Weinberg, Marcelo O Grecco, Gimena S Ferro, Debora J Seigelshifer, Nancy V Perroni, Francisco J Terrier, Analía Sánchez-Luceros, Esteban Maronna, Ricardo Sánchez-Marull, Isabel Frahm, Marcelo D Guthmann, Daniela Di Leo, Eduardo Spitzer, Graciela N Ciccia, Juan Garona, Marina Pifano, Ana V Torbidoni, Daniel E Gomez, Giselle V Ripoll, Roberto E Gomez, Ignacio A Demarco, Daniel F Alonso

**Affiliations:** Gynecology Service, Anesthesiology Service, Allergy and Immunology Unit and Central Laboratory, ‘Eva Peron’ Hospital, San Martín, Argentina; Breast Pathology Unit, Italian Hospital, La Plata, Argentina; Thrombosis and Hemostasis Department, National Academy of Medicine, IMEX-ANM, Buenos Aires, Argentina; Pathology Service, Mater Dei Sanatorium, Buenos Aires, Argentina; Elea Laboratories, Buenos Aires, Argentina; Chemo-Romikin, Buenos Aires, Argentina; Laboratorio de Oncología Molecular, Universidad Nacional de Quilmes, R. Sáenz Peña 352, Bernal, B1876BXD Buenos Aires, Argentina

**Keywords:** dDAVP, Surgery, Hemostasia, von Willebrand factor, Circulating tumor cells, Breast cancer trial

## Abstract

Desmopressin (dDAVP) is a well-known peptide analog of the antidiuretic hormone vasopressin, used to prevent excessive bleeding during surgical procedures. dDAVP increases hemostatic mediators, such as the von Willebrand factor (vWF), recently considered a key element in resistance to metastasis. Studies in mouse models and veterinary trials in dogs with locally-advanced mammary tumors demonstrated that high doses of perioperative dDAVP inhibited lymph node and early blood-borne metastasis and significantly prolonged survival. We conducted a phase II dose-escalation trial in patients with breast cancer, administering a lyophilized formulation of dDAVP by intravenous infusion in saline, 30–60 min before and 24 h after surgical resection. Primary endpoints were safety and tolerability, as well as selection of the best dose for cancer surgery. Secondary endpoints included surgical bleeding, plasma levels of vWF, and circulating tumor cells (CTCs) as measured by quantitative PCR of cytokeratin-19 transcripts. Only 2 of a total of 20 patients experienced reversible adverse events, including hyponatremia (grade 4) and hypersensitivity reaction (grade 2). Reactions were adequately managed by slowing the infusion rate. A reduced intraoperative bleeding was noted with increasing doses of dDAVP. Treatment was associated with higher vWF plasma levels and a postoperative drop in CTC counts. At the highest dose level evaluated (2 μg/kg) dDAVP appeared safe when administered in two slow infusions of 1 μg/kg, before and after surgery. Clinical trials to establish the effectiveness of adjunctive perioperative dDAVP therapy are warranted. This trial is registered on www.clinicaltrials.gov (NCT01606072).

## Background

Desmopressin (1-deamino-8-d-arginine vasopressin or dDAVP) is a peptide analog of the naturally occurring human antidiuretic hormone, vasopressin. It was first synthesized by Zaoral et al. (1967), being a selective agonist for the vasopressin V2 cell membrane receptor (V2R) present in kidney tubules and endothelia of blood vessels. Activation of endothelial V2R by dDAVP causes cAMP-mediated signaling followed by the release of von Willebrand factor (vWF), coagulation factor VIII and tissue-type plasminogen activator into the blood (Juul et al. 2014). The hemostatic effects of dDAVP at doses as low as 0.2–0.3 µg per kg of body weight make it an often-used treatment for the management of bleeding disorders, and is also being evaluated as a blood-saving agent in surgery or trauma (Mannucci 1997; Svensson et al. 2014). The evidence for elevated vWF levels as a risk factor for venous thromboembolism is weak and dDAVP appears safe for perioperative use. The compound has few side effects but it is recommended caution in small children and elderly, due to the risk of fluid retention and hyponatremia after repeated administration (Svensson et al. 2014).

Beyond its role in hemostasis, vWF has emerged as a pivotal regulator of tumor cell metastasis. Using a vWF-deficient mouse model, it was demonstrated that vWF plays a protective role against tumor cell dissemination in vivo by inducing apoptosis of metastatic cells, presumably early after their arrest in the microvasculature of the target organ (Terraube et al. 2006, 2007). Interestingly, aggressive human breast cancer cells expressing high levels of ADAM28 (a disintegrin and metalloproteinase 28) are capable of avoiding vWF-induced apoptosis in the circulatory system at micrometastatic sites. ADAM28 specifically binds to vWF and renders it inactive by cleaving, thus favoring the survival of metastatic cells (Mochizuki et al. 2012). Since physiological levels of vWF can induce cancer cell apoptosis, an attractive strategy could be to stimulate endothelial secretion of vWF by a pharmacological intervention, such as dDAVP infusion, aimed at increasing host resistance to metastasis (Ripoll and Alonso 2013).

Vasopressin receptors have been detected in many human cancer cell lines (Petit et al. 2001), including breast cancer (North et al. 1995, 1999), and it is known that dDAVP exerts some direct antiproliferative effect against V2R-expressing human breast carcinoma cells (Keegan et al. 2006). Such action is mediated through agonist V2R signaling, involving activation of adenylate cyclase with consequent intracellular cAMP elevation and protein kinase A activation. The cytostatic effect could be blocked by the selective nonpeptide V2R antagonists satavaptan (Keegan et al. 2006) and tolvaptan (Iannucci et al. 2011). In mouse mammary tumor models, intravenous (IV) administration of dDAVP prevented the development of blood-borne metastases (Alonso et al. 1999), and also decreased axillary lymph node involvement when administered at high doses during manipulation and surgical removal of the primary tumor (Giron et al. 2002). In addition, more recent studies in human and mouse mammary cancer cells have found that dDAVP can induce anti-angiogenic effects associated with the proteolytic conversion of plasminogen to angiostatin (Ripoll et al. 2013).

A pilot veterinary clinical trial in dogs with locally-advanced mammary cancer showed that a perioperative infusion of dDAVP at high doses of 1 μg/kg significantly prolonged disease-free and overall survival (Hermo et al. 2008). It seems that dDAVP infusion during the surgical phase not only inhibits perioperative metastatic events but also combats micrometastases that occurred before surgery. An extended veterinary trial confirmed these observations, demonstrating a reduced incidence of local relapses and lung metastasis in perioperatively treated animals, and a particular survival benefit in cases with high-grade carcinoma (Hermo et al. 2011).

Considering the well-known hemostatic effect and tolerability of dDAVP as well as its potential antimetastatic properties, we conducted a phase II dose-escalation trial in patients with breast cancer, administering a lyophilized formulation of dDAVP by IV infusion in saline, before and after surgical resection of primary tumor.

## Patients and methods

### Patients

Patients were enrolled from the “Eva Peron” Hospital, San Martin and the Italian Hospital, La Plata (Argentina). Eligible patients were otherwise healthy women between 18 and 65 years of age, with histological and/or cytological diagnosis of breast carcinoma (Stage 0, I, II) and managed by mastectomy or lumpectomy as primary treatment, including sentinel lymph node biopsy. Exclusion criteria included pregnancy or breast-feeding, hormonal treatment, known hypersensitivity to dDAVP or vasopressin, severe von Willebrand’s disease or hemophilia, syndrome of inadequate secretion of antidiuretic hormone, renal impairment or hyponatremia, congestive heart failure, blood hypertension, heart arrhythmia, thromboembolic disease, diabetes type I or II, any underlying coronary disease detected in pre-surgical evaluations, symptoms or evidence of metastasis on images and other malignant diseases. All patients provided written informed consent. The study was approved by the ethics committee at each site and by the National Administration of Drugs, Food and Medical Technology (ANMAT) in Argentina (No. NCT01606072).

### Study design

This was an open-label, dose-escalation phase II trial. Primary endpoints were safety and tolerability in breast cancer patients undergoing surgery as first treatment, as well as selection of the best dose of dDAVP for perioperative use in oncology. Secondary endpoints included surgical bleeding, plasma levels of vWF, and circulating tumor cells (CTCs).

### Perioperative administration of study treatment and anesthesia

Eligible patients were administered with dDAVP divided into 2 IV infusions, the first started preoperatively 30–60 min before surgery and the second postoperatively 24 h later. A lyophilized formulation of dDAVP (Surprex TM, Elea Laboratories, Buenos Aires, Argentina) was diluted in 100 mL of saline solution and slowly infused over the course of approximately 20–30 min. Five groups of at least four patients each received increasing total dDAVP doses of 0.5, 1.0, 1.25, 1.5 and 2.0 μg/kg, according to the scheme in Table 1. If no dose-limiting toxicity occurred, dosages were escalated to the next cohort of patients.

**Table 1:** Treatment groups, dosage and schedule of administration of perioperative dDAVP

Group	First dose (µg/kg) 30–60 min before surgery	Second dose (µg/kg) 24 h after surgery	Total dose (µg/kg)
1	0.25	0.25	0.5
2	0.5	0.5	1.0
3	0.75	0.5	1.25
4	1.0	0.5	1.5
5	1.0	1.0	2.0

Anesthesia was induced and maintained by target-controlled infusion of remifentanil (1–4 μg/kg) and propofol (1.5 mg/kg). Vecuronium bromide (0.1 mg/kg) was administered for muscle relaxation and endotracheal intubation. All patients were pre-oxygenated for 3 min with 100 % oxygen using face mask ventilation.

### Safety assessments

Safety and tolerability were assessed for all enrolled patients from the time the patient signs the informed consent through post-treatment follow-up. Adverse events were graded according to the NCI Common Toxicity Criteria for Adverse Events (CTCAE, Version 4.0). Serious adverse events were reported to the sponsor and the ethics committees and were followed up until resolution.

### Biochemical analysis

Blood was drawn within 7 days prior to surgery to obtain a baseline, and postoperatively 90–120 min after the first and the second dose of dDAVP. All laboratory assays were performed by investigators blinded to the clinical data, as described elsewhere (Sanchez-Luceros et al. 2010). The vWF antigen (vWF:Ag) was measured by ELISA. The functional activity of vWF was analyzed by the von Willebrand ristocetin cofactor (vWF:RCo) assay using formalin-fixed platelets. The factor VIII levels (FVIII:C) were assayed applying the one-stage method. The standard pool was periodically calibrated against the WHO International Standard for FVIII and vWF in plasma (07/316).

### Quantitative real-time reverse transcription-PCR (qPCR) detection of CTCs

CTCs were measured by qPCR assay for expression of cytokeratin-19 (CK-19) mRNA in whole blood (Ring et al. 2005). Total RNA was purified from peripheral blood stored in guanidine thiocyanate (Promega, Madison, WI) using QuickZol reagent (Kalium Technologies, Buenos Aires, Argentina). DNase treatment was carried out using a DNase I amplification grade kit (Life Technologies, Breda, the Netherlands) according to the manufacturer’s instructions. The RNA pellets were dissolved in nuclease-free water and stored at −70 °C prior to use. RNA was reverse transcribed with SuperScript III first-Strand kit (Life Technologies) according to the manufacturer’s protocol. Real-time PCR was performed with SYBR Green PCR Master Mix (Life Technologies) and StepOne Real-Time PCR System (Applied Biosystems, Foster City, CA, USA). The following specific primers were used as described elsewhere (Ring et al. 2005): for CK-19, forward: 5′-TGC GGG ACA AGA TTC TTG GT-3′ and reverse: 5′-TCT CAA ACT TGG TTC GGA AGT CA-3′; for glyceraldehyde 3-phosphate dehydrogenase (GAPDH), forward: 5′-CAT GGG TGT GAA CCA TGA GA-3′ and reverse: 5′-CAG TGA TGG CAT GGA CTG TG-3′. All sample plates were run with positive controls (RNA from MCF-7 human breast cancer cell line) and no template negative controls. The following thermal cycling conditions were used: 48 °C for 30 min, 95 °C for 10 min, 40 cycles of 95 °C for 15 s followed by 60 °C for 60 s. Each sample was analyzed in triplicate and mean cycle threshold (Ct) values were used for further analysis. Ct values for CK-19 were normalized for GAPDH expression levels and expressed in relation to positive control samples. Relative quantification (RQ) values were calculated as 2^−ΔΔCt^.

### Immunohistochemical detection of V2R

Breast tumor samples were fixed in 10 % formalin, embedded in paraffin, and tissue sections of 4 µm were cut and placed on silane coated slides. Immunohistochemistry was performed on a Bond automated system (Leica Biosystems, Newcastle, UK). Sections were dewaxed and pretreated with the epitope retrieval solution 2 (EDTA buffer, pH 8.8) at 100 °C for 20 min. Immunostaining was carried out using polyclonal rabbit antibodies against the human V2R (V5514; 1:100 dilution, Sigma-Aldrich) at room temperature for 20 min, and a biotin-free, polymeric horseradish peroxidase (HRP)-linked antibody conjugate as a secondary antibody. Sections were counterstained with hematoxylin. Kidney tubules, as well as V2R-expressing MCF-7 human breast cancer xenografts generated in nude mice (Garona et al. 2015), were used as positive controls for V2R expression.

### Statistical analysis

PRISM 6, Version 6.01 (GraphPad Software Inc, La Jolla, CA, USA) was used to conduct all statistical analyses. P values less than 0.05 were considered statistically significant. For multiple group comparisons one-way or two-way ANOVA, followed by Tukey post hoc test were applied after normal distribution of data was confirmed using the Shapiro–Wilk normality test. In addition, the homoscedasticity was determined with Bartlett’s test. For non-normally distributed data or when homoscedasticity was not supported, Kruskal–Wallis test was performed. The cut-off value for CK-19 mRNA was determined with receiver operating characteristics (ROC) curve analysis (minimal false-negative and false-positive results).

## Results

The trial accrued a total of 21 patients from April 2012 to February 2014. One patient who developed a hypertensive episode during the night before surgery was ineligible and excluded from the study. Characteristics of the enrolled patients are summarized in Table 2. Among the 20 patients evaluable for toxicity, adverse events attributable to dDAVP were observed in two patients and all were reversible. Laboratory examinations of one patient included in treatment group 3 (1.25 μg/kg) showed hyponatremia (serum sodium levels <120 mEq/L, grade 4) 1 h after the first dDAVP dose. The patient also experienced nausea and mild dyspnea (grade 1). These events were considered non-serious as they were transient and reversible, and hyponatremia was spontaneously corrected 24 h later (137 mEq/L). Another patient of treatment group 4 (1.5 μg/kg) showed signs of a hypersensitive reaction early after starting the preoperative dDAVP infusion, manifesting hot flushing, skin rash and palpitations (grade 2). This event was considered serious and treatment was interrupted before completion of the first dose. Patient was medicated IV with diphenhydramine (20 mg) and dexamethasone (8 mg), showing complete resolution of symptoms within 45 min. Since treatment was interrupted, this patient was not evaluable for secondary endpoints. Reactions were adequately managed by slowing the infusion rate of dDAVP to 30–40 min in treatment group 5 (2 μg/kg). The maximum tolerated dose was not reached, and the individual dose of 1 μg/kg given preoperatively and postoperatively was then considered for further studies. Median follow-up was 24 months (range 17–39 months). None of the patients relapsed during follow-up.

**Table 2:** Descriptive characteristics of patients enrolled in the study (n = 20)

Patient characteristic	No.
Age, median (range)	47 years (36–62)
Tumor size, median (range)	20 mm (5–40)
Histopathology	
Ductal carcinoma in situ (DCIS)	4 (20 %)
Invasive ductal carcinoma	15 (75 %)
Invasive lobular carcinoma	1 (5 %)
Axillary involvement in invasive carcinoma, n = 16	7 (43 %)
Molecular subtype of invasive carcinoma, n = 16	
Luminal (A and B)	11 (69 %)
Her2	1 (6 %)
Triple negative	4 (25 %)
V2R status known, n = 18	
Positive tumor expression	6 (33 %)
Type of surgery	
Breast conserving surgery	12 (60 %)
Mastectomy	8 (40 %)
Postoperative adjuvant therapy	
None (DCIS)	4 (20 %)
Cyclophosphamide-based chemotherapy	6 (30 %)
Radiotherapy	3 (15 %)
Chemoradiotherapy	7 (35 %)

**Fig. 1:**
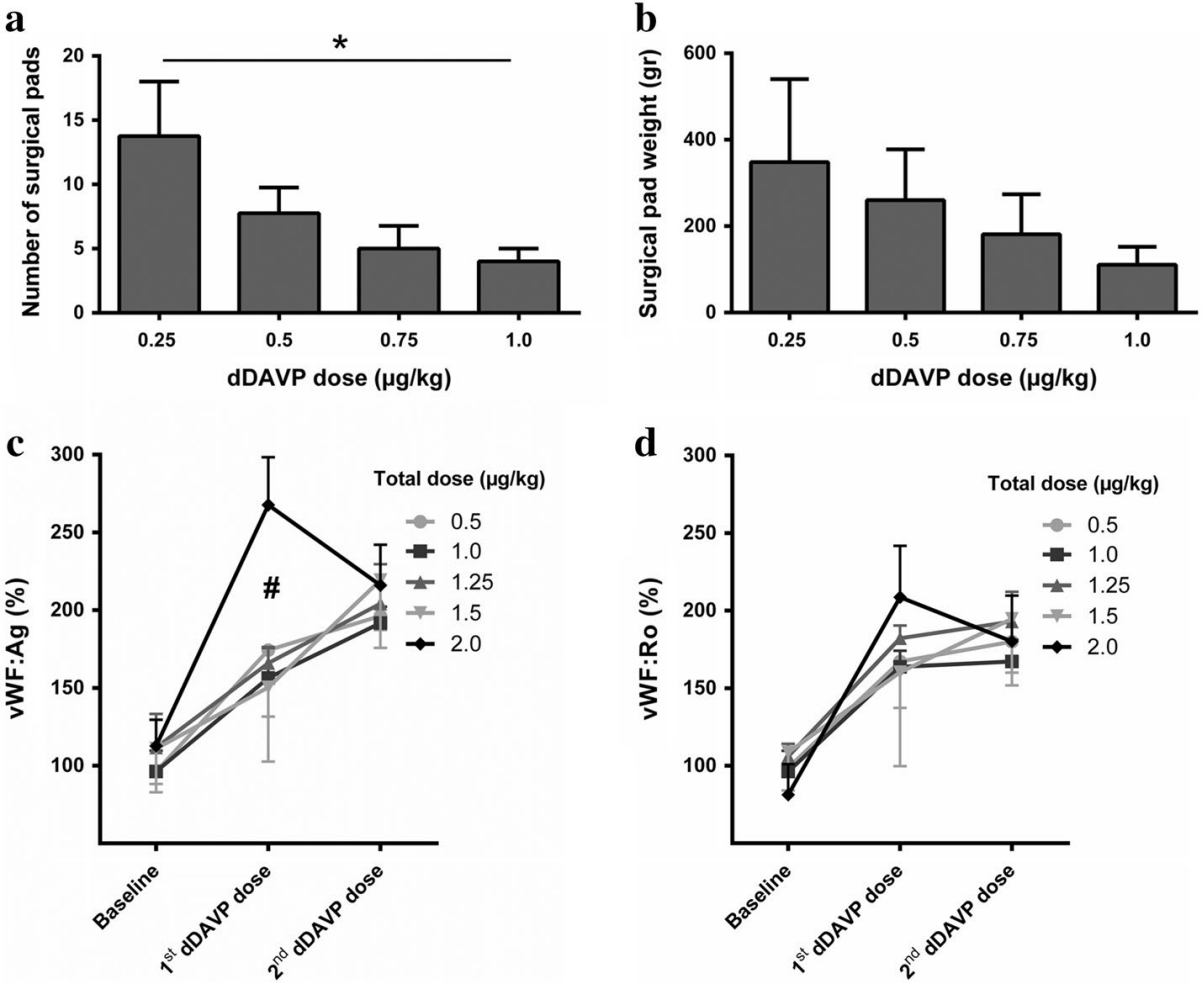
Hemostatic effects of perioperative dDAVP. **a** Number and **b** weight of surgical pads used during the surgical procedure, as a function of the preoperative first dose of dDAVP (treatment groups 4 and 5 are presented together, since in both cases received 1 µg/kg). *p < 0.05 (1.0 versus 0.25 µg/kg), ANOVA with Tukey post-test. **c** vWF antigen (vWF:Ag) and **d** functional vWF (vWF:RCo) levels in samples collected prior to surgery (*baseline*), and after the preoperative dose (1st dDAVP dose) and the postoperative dose (2nd dDAVP dose). ^#^p < 0.05 (2.0 µg/kg versus all other dose levels), two-way ANOVA with Tukey post-test. In all cases, data represent mean ± SEM.

A reduced intraoperative bleeding of up to 50 % was noted with increasing doses of dDAVP, as measured by the number or weight of pads used during surgical procedure (Fig. 1a, b). A significant reduction was observed in the number of surgical pads used in patients receiving a preoperative first dose of dDAVP of 1 μg/kg (treatment groups 4 and 5, considered together) in comparison to lower doses (see also Fig. 1a). As expected, vWF:Ag plasma levels exhibited a mean increase of 50–100 % with respect to baseline after each preoperative and postoperative dDAVP infusion, and maximum levels were obtained in patients of group 5 treated with the highest total dose of 2 μg/kg (Fig. 1c). Similar results were found for vWF:RCo (Fig. 1d) and FVIII:C levels (data not shown).

**Fig. 2:**
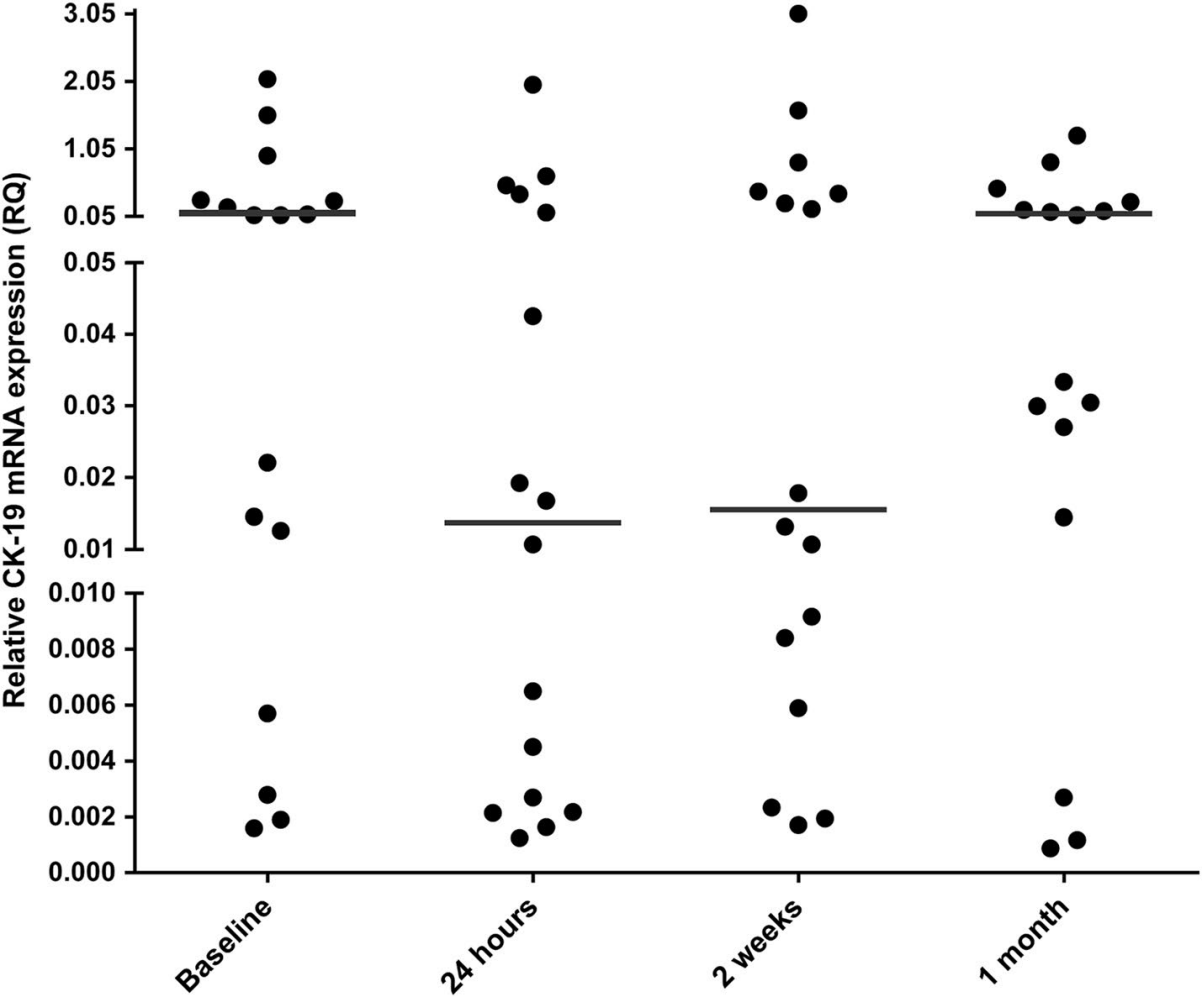
Detection of circulating tumor cells (CTCs) by qPCR. CTCc were assessed by means of expression of transcripts for CK-19 in whole blood, as described in detail in “Patients and methods”. Samples from 16 patients were obtained within 7 days prior to surgery (*baseline*), and 24 h, 2 weeks and 1 month after surgery. Data from all treatment groups were pooled. *Horizontal lines* indicate the median values. The cut-off RQ value was 0.00445 for healthy woman volunteers aged 25–61 years, based on ROC analysis (specificity: 100 %; sensibility: 81.25 %; area = 0.91).

Evaluable samples were available for CTCs assessment from 16 of the 20 patients enrolled. A preliminary analysis indicated no significant differences between treatment groups, and thus data were pooled together due to the small number of patients. Detectable levels of CK-19 mRNA were found in several patients, and 9 of the 16 patients had high RQ values of >0.05 before surgery at baseline (Fig. 2). Twenty-four hours after surgery, only 5 patients showed high RQ values and also the median levels of expression were reduced. Two weeks later, median values remained reduced, but returned to baseline 1 month after surgery.

**Fig. 3:**
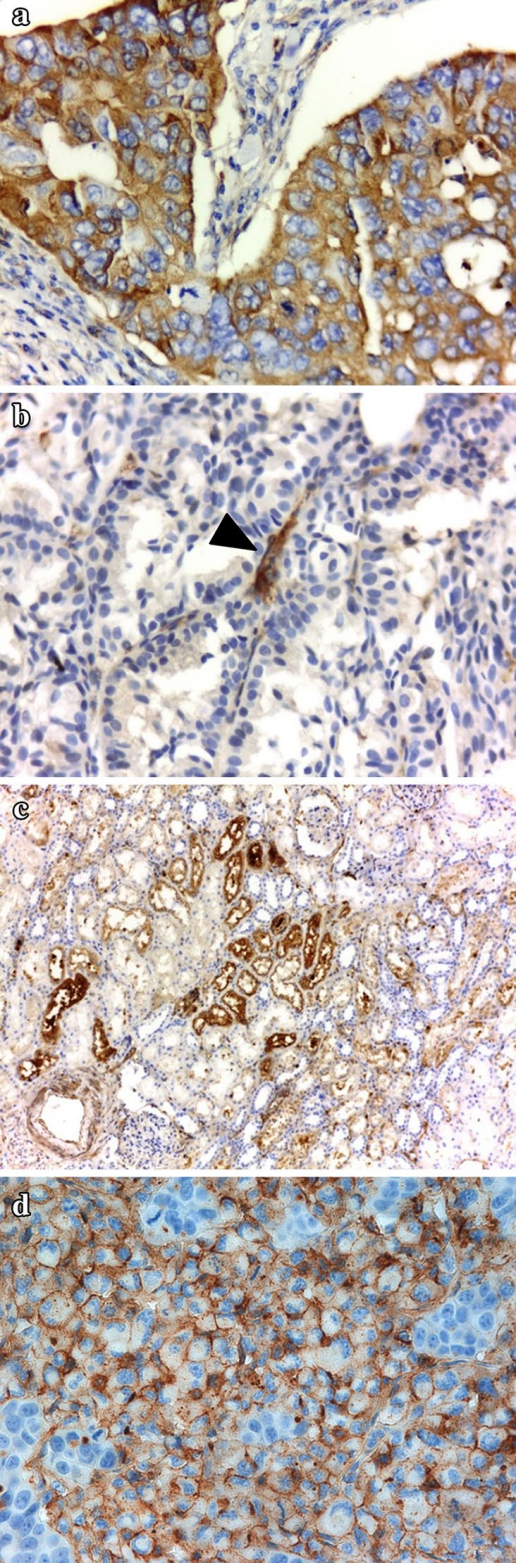
Immunohistochemical staining of vasopressin receptors. V2R expression was detected using polyclonal antibodies against the human receptor, as described in detail in “Patients and methods”. Representative pictures of tumor sections from patients enrolled in the trial and positive control tissue are depicted. **a** Breast carcinoma expressing V2R **b** V2R-negative breast carcinoma **c** Kidney tubules **d** MCF-7 human breast carcinoma xenograft. Arrowhead denotes positive staining of small vessels. Original magnification: **a**, **b**, **d** ×400; **c** ×100.

We examined the expression of V2R by immunohistochemistry in paraffin tumor samples available from 18 patients. In all cases, V2R was detected in endothelial cells of small vessels of tumor stroma or surrounding tissues. Six of the 18 cases evaluated revealed positive expression of V2R in breast carcinoma cells (Fig. 3; see also Table 2). Expression pattern was cytoplasmic, either diffuse or focal, with membrane accentuation, and the intensity of staining ranged from moderate to strong.

## Discussion

Pioneer works by Mannucci et al. (1977, 1981) in healthy subjects and patients with hemophilia A and von Willebrand´s disease demonstrated a good tolerance and efficacy of dDAVP as a hemostatic agent at doses up to 0.5 μg/kg by the IV route. However, since single doses of 0.2–0.3 μg/kg seemed to produce a near-maximal response in healthy subjects a reduction in dosage was suggested, in order to reduce side effects such as tachycardia (Mannucci et al. 1981). There are anecdotal case reports that document the satisfactory perioperative use of dDAVP in oncology patients with hemostatic disorders, including a case of a woman with Glanzmann thrombasthenia receiving 0.4 μg/kg of the compound during the resection of a breast tumor later diagnosed as fibroadenoma (Ohishi et al. 1990).

To our knowledge, this is the first dose-escalation trial of dDAVP as a perioperative adjunctive treatment in the management of operable cancers. The compound was well tolerated at the highest total dose level tested in this study (2 μg/kg) when administered divided in two slow IV infusions of 1 μg/kg, 30–60 min before and 24 h after surgery. Two patients developed adverse events, including hyponatremia and a hypersensitivity reaction that were completely reversible. It is known that the hemostatic dosage is higher than the dose used for antidiuresis. Maximal antidiuretic effect is already achieved with low doses, while duration of hemostatic effect tend to prolong with increasing doses (Lethagen et al. 1998). Although water retention is not a prominent clinical problem, the risk of hyponatremia should be taken into account, particularly in elderly patients receiving hypotonic solutions or after frequently, repeated doses of dDAVP (Svensson et al. 2014; Lethagen et al. 1998).

Intraoperative bleeding can be a major risk for gastrointestinal or urologic cancers, but it is not a serious problem in early-stage breast cancer patients as included in this study. However, dDAVP still significantly reduced blood loss at a preoperative dose of 1 μg/kg (treatment groups 4 and 5) as determined by surgical pads used during operation. In this sense, a significantly higher increase of vWF was also noted at the highest dose level with respect to the other treatment groups. Interestingly, vWF is now considered as a versatile multifunctional protein (Rauch et al. 2013) given its potential role in different non-hemostatic processes, like metastasis resistance and tumor cell apoptosis (Terraube et al. 2006, 2007). It is known that interaction of vWF with metastatic cells is mediated via integrin αVβ3, affecting their adhesion and survival. However, certain aggressive cancer cells are able to escape vWF-induced cell death through production of the protease ADAM28 that can counterbalance the pro-apoptotic function of vWF (Mochizuki et al. 2012).

Preclinical studies in aggressive mouse tumor models (Alonso et al. 1999; Giron et al. 2002) and veterinary clinical trials in dogs with locally-advanced mammary cancer (Hermo et al. 2008, 2011) have demonstrated inhibition of metastatic progression and survival benefit, respectively, of perioperative dDAVP at doses in the range of 1–2 μg/kg. In the present clinical trial, CTCs were evaluated at different times after perioperative dDAVP treatment, as measured by qPCR detection of CK-19 transcript in peripheral whole blood. An important proportion of blood samples from breast cancer patients were positive for CK-19 preoperatively at baseline, but median expression levels were reduced early postoperatively and also 2 weeks later. One month after surgery, CTCs returned to baseline levels. Even though no placebo or control group underwent surgery was studied, it is noteworthy that surgical manipulation of breast cancer has been consistently associated with a postoperative increase of CK-19 mRNA-positive cells in peripheral blood (Daskalakis et al. 2011; Galan et al. 2002). Thus, it seems that administration of dDAVP during the perioperative period not only improves hemostatic control but also appear to minimize shedding and/or survival of breast carcinoma cells.

Recent findings indicated that dDAVP is able to reduce tumor angiogenesis by inducing the formation of angiostatin (Ripoll et al. 2013), a naturally occurring inhibitor of angiogenesis generated by limited proteolysis of plasminogen. V2R-expressing breast cancer cells are stimulated by dDAVP to secrete plasminogen activators such as urokinase, thus excising angiostatin from plasminogen. Biological effects of the peptide on both tumor and endothelial cells appear complex and required further investigations. Notwithstanding, perioperative administration of dDAVP seems to induce a dual angiostatic and antimetastatic effect, breaking cooperative tumor-endothelium interactions in incipient metastatic lesions (Garona and Alonso 2014). Here we explored the expression of V2R in breast cancer tissues by immunohistochemistry, finding one-third of cases were positive and thus may respond with this full dual action. The negative cases, however, still would benefit from dDAVP treatment through endothelial vWF secretion with consequent hemostatic and antimetastatic effects. Furthermore, experimental evidence has suggested a direct role of vWF in the modulation of angiogenesis. Inhibition of vWF by short interfering RNA in endothelial cells caused increased in vitro angiogenesis and an enhanced vascularization response was observed in vWF-deficient mice (Starke et al. 2011).

In conclusion, at the highest dose level evaluated perioperative dDAVP appeared to be safe when administered in two slow IV infusions of 1 μg/kg, before and after the surgical procedure. The results of our study suggest that treatment is associated with reduction of intraoperative bleeding, higher circulating vWF levels and a drop in CTC counts after surgery. Perioperative or early postoperative therapies should target not only circulating or residual cancer cells, but also the wound healing mechanisms usurped by these cells to survive and metastasize (Harless 2009). In this regard, the perioperative period is an underutilized window of opportunity, where tumor-host interactions can be modulated to reduce the risk of local relapses and metastases (Coffey et al. 2003). We believe that the present study provides promising evidence to improve the outcome of breast cancer surgery using a well-known hemostatic agent with good tolerance. Clinical trials to establish the effectiveness of administering adjunctive perioperative dDAVP therapy are warranted.
